# Tumor-Derived Factors and Reduced p53 Promote Endothelial Cell Centrosome Over-Duplication

**DOI:** 10.1371/journal.pone.0168334

**Published:** 2016-12-15

**Authors:** Zhixian Yu, Kevin P. Mouillesseaux, Erich J. Kushner, Victoria L. Bautch

**Affiliations:** 1 Curriculum in Genetics and Molecular Biology, The University of North Carolina at Chapel Hill, Chapel Hill, North Carolina, United States of America; 2 Department of Biology, The University of North Carolina at Chapel Hill, Chapel Hill, North Carolina, United States of America; 3 McAllister Heart Institute, The University of North Carolina at Chapel Hill, Chapel Hill, North Carolina, United States of America; University of Illinois at Chicago, UNITED STATES

## Abstract

Approximately 30% of tumor endothelial cells have over-duplicated (>2) centrosomes, which may contribute to abnormal vessel function and drug resistance. Elevated levels of vascular endothelial growth factor A induce excess centrosomes in endothelial cells, but how other features of the tumor environment affect centrosome over-duplication is not known. To test this, we treated endothelial cells with tumor-derived factors, hypoxia, or reduced p53, and assessed centrosome numbers. We found that hypoxia and elevated levels of bone morphogenetic protein 2, 6 and 7 induced excess centrosomes in endothelial cells through BMPR1A and likely via SMAD signaling. In contrast, inflammatory mediators IL-8 and lipopolysaccharide did not induce excess centrosomes. Finally, down-regulation in endothelial cells of p53, a critical regulator of DNA damage and proliferation, caused centrosome over-duplication. Our findings suggest that some tumor-derived factors and genetic changes in endothelial cells contribute to excess centrosomes in tumor endothelial cells.

## Introduction

Tumor progression requires angiogenesis, a hallmark of cancer development, and tumor vessels enable tumor metastasis by providing a conduit for tumor cell invasion and spread [1, 2]. Although tumor vessels are a critical part of the tumor micro-environment, anti-angiogenic therapies have had no effect or provided transitory improvement, indicating that tumor vessels become resistant to angiogenesis inhibitors [3]. Consistent with the lack of effectiveness of anti-angiogenic therapy, recent studies show that endothelial cells (EC) that line tumor vessels have genetic abnormalities such as aneuploidy. Aneuploidy is often associated with excess centrosomes, and up to 30% of tumor EC have excess centrosomes [4–6]. Centrosomes form the microtubule-organizing center (MTOC) in interphase cells to regulate cell migration, polarity, and adhesion, and they form the spindle poles that segregate chromosomes during mitosis [7]. Thus tumor EC acquire permanent structural and genetic alterations via excess centrosomes that likely contribute to the phenotypic and functional abnormalities of tumor blood vessels.

Tumor blood vessels are thought to arise from normal vessels that enter the tumor [8, 9], suggesting that the environment is responsible for inducing excess centrosomes in EC. Tumor cells secrete elevated levels of various growth factors [10], and our previous work showed that elevated levels of vascular endothelial growth factor A (VEGF-A) induce centrosome over-duplication in EC [11]. However, the frequency of centrosome over-duplication in tumor-derived EC is significantly higher than that induced by excess VEGF-A [6, 11]. Thus other up-regulated signaling pathways in the tumor environment likely contribute to centrosome over-duplication in EC. For example, bone morphogenetic protein (BMP), which is required for appropriate angiogenesis, is up-regulated in certain cancers [12]. Furthermore, different BMP ligands such as BMP2, BMP4, BMP6 and BMP7 induce angiogenesis [13], and BMP2 and BMP4 promote tumor angiogenesis [13].

In addition to growth factors, the tumor environment is hypoxic and has elevated levels of inflammatory cytokines. The tumor environment is hypoxic in part because of abnormal tumor blood vessels [14]. Hypoxia activates the hypoxia-inducible factor (HIF) family of transcription factors, which further induce expression of numerous downstream targets, including VEGF-A [15]. Inflammation is also a hallmark of the tumor environment and is thought to promote tumor growth [16], perhaps via secretion of angiogenic chemokines such as Interleukin 8 (IL-8) that induce tumor angiogenesis [17]. It is not known whether hypoxia or inflammation promote excess centrosomes in EC.

In this report, we analyzed the effects of specific inputs elevated in the tumor environment on centrosome over-duplication in EC. We found that elevated levels of some BMP ligands are sufficient to induce centrosome over-duplication in EC, using BMP receptor 1A and likely via downstream SMAD signaling. Additionally, hypoxia promoted EC centrosome over-duplication through a VEGF-A-independent mechanism. In contrast, inflammatory mediators did not affect centrosome numbers in EC. In addition to environmental factors, down-regulation of the tumor-suppressor p53 induced centrosome over-duplication in EC. These results indicate that both environmental and genetic factors contribute to centrosome over-duplication in EC, and may contribute to the high frequencies seen in tumor vessels.

## Materials and Methods

### Cell culture

Human umbilical vein endothelial cells (HUVEC, Lonza Group cc-2519), human brain microvascular endothelial cells (HBMEC, Cell Systems ACBRI 376) and human umbilical artery endothelial cells (HUAEC) were cultured in endothelial growth medium-2 (EGM-2, Lonza Group cc-3162). Human lung microvascular endothelial cells (HMVEC-L, Lonza Group cc-2527) were cultured in EGM-2 MV (Lonza Group cc-3102). Normal mouse EC (NEC) were originally isolated from mouse mammary glands and cultured in EGM-2 [6]. Growth factors or lipopolysaccharide (LPS, List Biological Laboratories 201) were added to cultures at indicated concentrations. Exogenous recombinant growth factors used in this study were VEGFA-165 (PeproTech 100–20), BMP2 (R&D Systems 355-BM-010), BMP4 (R&D Systems 314-BP-010), BMP6 (R&D Systems 507-BP-020), BMP7 (R&D Systems 354-BP-010), and Interleukin-8 (IL-8, PeproTech 200–08). VEGF-A and BMP were used at 200 ng/ml, and IL-8 was added at indicated concentrations. Culture medium was replaced daily for 4 days, and cells were maintained at 30–70% confluence. To study signaling, HUVEC were cultured in Opti-MEM for 4 hr before treatment with 200 ng/ml BMP ligands in Opti-MEM for 30 min. To validate the specificity of the HIF1α antibody, HUVEC were treated with 100 μM CoCl_2_ for 4 hr in EGM-2 prior to fixation and staining.

Lipofectamine RNAiMAX (Life Technologies 13778–150) was used for siRNA transfection according to manufacturer protocols. siRNAs were: non-targeting siRNA (Life technologies 4390847), BMPR1A siRNA (Life technologies 4392420-s280), BMPR1B siRNA (Life technologies 4392420-s2043) and BMPR2 siRNA (Life technologies 4390824-s2046).

For hypoxia experiments, HUVEC were cultured in a hypoxia incubator flushed with 2% or 3% O_2_ for 4 days. The hypoxia incubator digitally sets the percentage of O_2_ at user-defined levels, and automatically controls the level of O_2_ by modulating N_2_ levels, which is supplied through a nitrogen air tank. 1 μg/ml of recombinant human VEGF Receptor-1 (Flt-1)/Fc (R&D Systems 321-FL-050) was added to medium to block VEGF-A signaling [18], and the medium was changed daily. In general, EC were immediately fixed with cold MeOH after hypoxic incubation. To test for translocation of HIF1α, EC were recovered in normoxia for 30 min before fixation. Hypoxic-mimetic agent desferrioxamine (DFO) and a hypoxia incubator chamber were kindly provided by Dr. Kimryn Rathmell.

### Immunofluorescence and microscopy

HUVEC were fixed in ice cold 100% MeOH for 10 min, then stained as previously described [19]. Briefly, fixed cells were blocked in 5% bovine serum in PBS for 1hr at room temperature (RT), then incubated with mouse anti-human γ-tubulin (1:5000, Sigma-Aldrich T6557), rabbit anti-human pericentrin (1:5000, Abcam ab4448), rabbit anti-human pSmad1/5 (1:500, Cell Signaling 9516) or mouse anti-human HIF1α (1:50, Novus biologicals NB100-105) at 4°C overnight. To validate HIF1α antibody specificity, fixed HUVEC were incubated with staining solution at 4°C overnight. After washing 3X 5 min in PBS, cells were incubated with secondary antibodies (1:250), including goat-anti-mouse Alexa 488 (Invitrogen A11029) or goat-anti-mouse Alexa 594 (Invitrogen A11005), and DRAQ7 (1:1000, Abcam ab109202) or SYTOX green (1:50,000, Invitrogen S7020), for 2hr at RT. Both primary and secondary antibodies were diluted in 5% bovine serum in PBS. Centrosome numbers in interphase endothelial cells were determined using a Zeiss LSM 5 Pascal microscope with a 100X objective. Mitotic cells were excluded using the DNA marker DRAQ7 in most experiments, and any evidence of nuclear envelope breakdown and/or chromosome condensation was used as exclusion criteria.

Nuclear pSMAD1/5 and HIF1α fluorescence intensities were quantified in ImageJ using a mask. Briefly, the DRAQ7 (nucleus) channel from compressed z-stacks was thresholded to segment nuclei and adjusted into a binary image. Intensity analysis was redirected from the binary image to the pSMAD1/5 or HIF1α channel by changing the “Set Measurements” parameter. “Analyze Particles” function was executed to determine pSMAD1/5 and HIF1α intensity in each nucleus.

### Western blot

Western blot analysis was performed as previously described, with slight modifications [11]. Briefly, HUVEC lysates were lysed using RIPA buffer supplemented with protease inhibitor (Cell Signaling 5871S). Proteins were separated on a 10% sodium dodecyl sulfate–polyacrylamide gel, transferred to a PVDF membrane (GE Healthcare, RPN303F), and blocked in 5% bovine serum albumin (BSA) in PBS with 1% tween-20 (Sigma P2287) for 1h at RT. Primary antibodies used were: rabbit anti-phospho-Smad1/5 (1:1000, Cell Signaling 9516), rabbit anti-Akt (1:1000, Cell Signaling 9272), rabbit anti-phospho-Akt (Ser473) (1:1000, Cell Signaling 4060), rabbit anti-phospho-ERK1/2 (Thr202/Tyr204) (1:1000, Cell Signaling 4370), rabbit anti-ERK 1/2 (1:1000, Cell Signaling 4695), mouse anti-HIF1α (1:500, Novus biologicals NB100-105), mouse anti-p53 (1:1000, Abcam ab1101) and rabbit anti-p53 (1:500, Abcam ab131442). Membranes were incubated with primary antibodies diluted in 1% BSA overnight at 4°C. Signal was detected with horseradish peroxidase (HRP) anti-rabbit (1:5000, Invitrogen G-21234) or HRP anti-mouse (1:30,000, Invitrogen 81–6720), and imaged via Clarity Western ECL Substrate (Bio-Rad 170–5061). Full original blots are shown (S6 Fig).

### Quantitative real-time PCR

HUVEC were collected 48 hr after siRNA transfection, and total RNA was isolated with TRIZOL (Life technologies 15596–026) according to the manufacturer’s protocol. 1 μg of RNA was used for synthesizing cDNA with iScript (Bio-Rad 1708891). cDNA products were diluted fivefold. For measuring BMPR1B, BMPR2 and GAPDH, 0.5 ul of diluted samples were used as templates; for BMPR1A, 5 ul of diluted samples were used. RT-PCR was preformed using iTaq universal SYBR Green supermix (Bio-Rad 1725121) in a 7900HT fast RT-PCR system (Applied Biosystems). Primer sequences were: GAPDH (forward: CCTCAAGATCATCAGCAATGCCTCCT; reverse: GGTCATGAGTCCTTCCACGATACCAA), BMPR1A (forward: AGCTACGCCGGACAATAGAA; reverse: CTATGACAACAGGGGGCAGT), BMPR1B (forward: GCCTGCCATAAGTGAGAAGC; reverse: CTTTCTTGGTGCCCACATTT), and BMPR2 (forward: GGTAAGCTCTTGCCGTCTTG; reverse: ATCTCGATGGGAAATTGCAG).

### Lentivirus infection

Human p53–targeted shRNA (clone ID: V3LHS_333920) with pGIPZ vector was obtained from Open Biosystems. Mouse p53–targeted shRNA clone (TRCN0000012360) with pLKO.1 vectors were obtained from the UNC Lenti-shRNA Core facility. The centrin-GFP-expressing lentiviral construct was previously generated [19]. Lentiviruses were made by the UNC Lenti-shRNA Core facility. Cells were infected with 100 μl/ml lentivirus in 5 ml medium plus 1μg/ml polybrene (Millipore) overnight at 37°C, then incubated for 4 days before fixation or collection. Virus lacking a target sequence (empty vector) was used as a control.

### Statistical analysis

The paired or unpaired two-tailed Student’s t-test was used to determine statistical significance in cases with 3 repeats. The Χ^2^ test was used to determine statistical significance in cases with 2 repeats. Error bars represent standard deviation from mean between experiments.

## Results

### Elevated levels of BMP ligands induce excess centrosomes in EC

**Fig 1:**
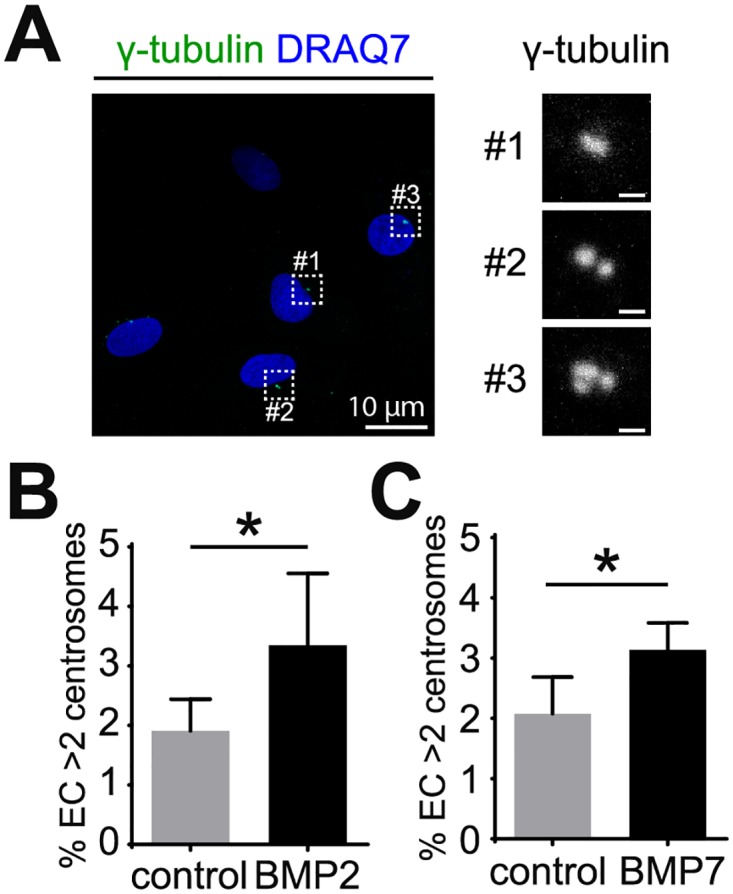
BMP2 and BMP7 induce excess centrosomes in EC. (A) Representative images of HUVEC with normal (#1 and #2) and over-duplicated centrosomes (#3). HUVEC were stained with γ-tubulin for centrosomes (green) and DRAQ7 for nuclei (blue). (B, C) Frequency of excess centrosomes in HUVEC after treatment with 200 ng/ml BMP2 (B) or BMP7 (C) for 4 days. Error bars, standard deviation from mean. Statistics: two-tailed unpaired Student’s t-test. *, p≤0.05. Scale bars: 1 μm unless indicated otherwise.

**Fig 2:**
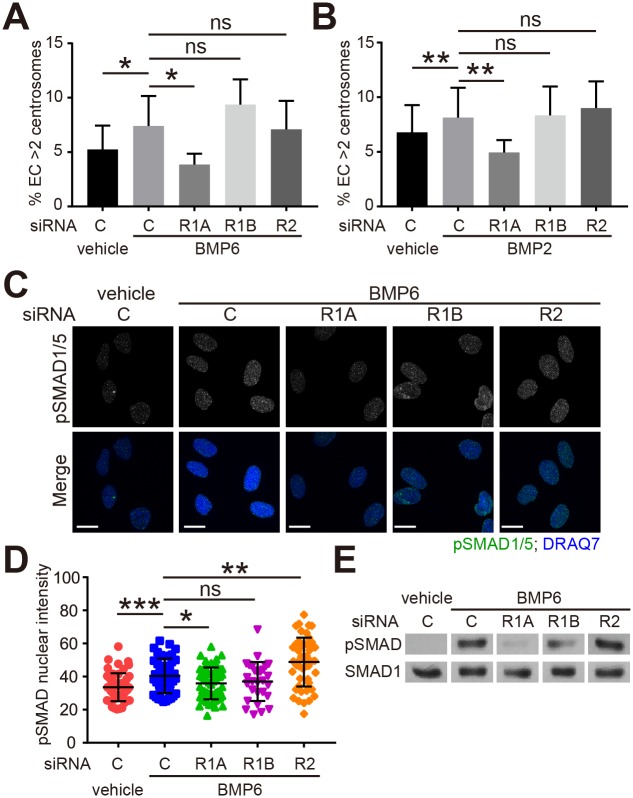
BMP-induced centrosome over-duplication is dependent on BMPR1A. (A, B) Frequency of excess centrosomes in indicated siRNA-treated HUVEC cultured with vehicle or 200 ng/ml of BMP6 (A) or BMP2 (B) for 4 days. C, non-targeting control siRNA; R1A, BMPR1A siRNA; R1B, BMPR1B siRNA; R2, BMPR2 siRNA. (C) Representative images of HUVEC treated with indicated siRNA and vehicle or BMP6 and stained for phospho-SMAD1/5 (pSMAD1/5, green) and nucleus (DRAQ7, blue). Cells were starved in Opti-MEM for 4 hr, followed by 30 min treatment with vehicle or BMP6. Only the nuclear pSMAD1/5 is shown (see Methods for details of mask). (D) Quantification of nuclear pSMAD1/5 in HUVEC treated as indicated. (E) Western blot of phospho-SMAD1/5 (pSMAD) and total SMAD1 in HUVEC treated as indicated. Cells were starved in Opti-MEM for 4 hr, then treated with vehicle or BMP6 for 30 min. Error bars, standard deviation from mean. Statistics: two-tailed paired (A, B) or unpaired (D) Student’s t-test. ns, not significant; *, p≤0.05; **, p≤0.01; ***, p≤0.001. Scale bars: 10 μm.

We began to dissect the different potential inputs to excess centrosome formation from the tumor environment by introducing elevated levels of different signaling pathways or by genetic manipulation of normal EC and assessing effects on centrosome over-duplication. Because BMP ligands regulate angiogenesis and are expressed in the tumor micro-environment, we asked whether elevated BMP signaling regulates centrosome number in EC. HUVEC treated with different BMP ligands were stained with anti-γ-tubulin antibodies to label centrosomes, and EC with different centrosome numbers were clearly identified (S1A Fig). Co-labeling with centrin-GFP and pericentrin revealed the same centrosome numbers, indicating that *de novo* centrosome over-duplication was scored (S1A Fig). As previously described, EC with 3 or more centrosomes were considered to have excess centrosomes (Fig 1A) [19]. Exposure to BMP2, BMP6, or BMP7 caused a significant increase in the percentage of HUVEC with excess centrosomes (Figs 1B, 1C and 2A). This effect was not observed with BMP4 treatment in HUVEC (S1B Fig), nor upon treatment with BMP2 or BMP6 in HUAEC, HBMEC or HMVEC-L (S1C–S1E Fig). These results indicate that some but not all BMP ligands induce excess centrosomes, and that different EC isolates respond differently to these ligands.

### BMP-induced centrosome over-duplication is BMP receptor type 1A (BMPR1A)-dependent

To understand the mechanism of BMP-induced centrosome over-duplication, we down-regulated BMP receptors in HUVEC. There are several BMP-specific receptors that include type 1A BMP receptor (BMPR1A/ALK3), type 1B BMP receptor (BMPR1B/ALK6), and type 2 BMP receptor (BMPR2) [20]. siRNA targeting of these three receptors efficiently and significantly knock-down their mRNA levels (S2A–S2C Fig). The increase in EC with excess centrosomes seen with BMP2 or BMP6 was blocked by BMPR1A knockdown, but not by BMPR1B or BMPR2 knockdown (Fig 2A and 2B). These findings suggest that BMPR1A is required for BMP-induced centrosome over-duplication.

Type 1 and type 2 BMP receptors form hetero-tetramers upon ligand binding that permits phosphorylation of downstream effectors called receptor-regulated SMAD (R-SMAD), including SMAD1 and SMAD5. Phosphorylated R-SMADs bind SMAD4 to translocate into the nucleus and modulate gene expression [20]. To further understand the mechanism of BMP-induced centrosome over-duplication, we examined the phosphorylation of SMAD1/5 by immunofluorescence. The levels of nuclear phospho-SMAD1/5 (pSMAD1/5) were significantly induced by BMP6 treatment in control siRNA, BMPR1B siRNA and BMPR2 siRNA-treated HUVEC, but not in BMPR1A siRNA-treated cells (Fig 2C and 2D), which was also confirmed by western blot (Fig 2E). These results suggest that BMPR1A is required for BMP-induced centrosome over-duplication through downstream R-SMAD activation.

### Inflammatory mediators do not promote excess centrosomes in EC

**Fig 3:**
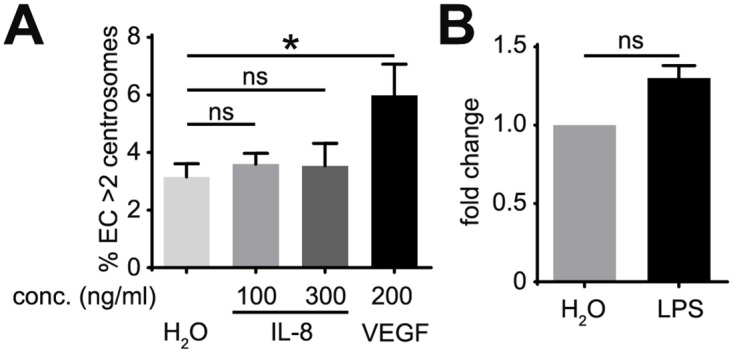
Inflammatory mediators do not induce excess centrosomes in EC. (A) Frequency of excess centrosomes in HUVEC after treatment with indicated factors for 4 days. (B) HUVEC incubated with 10 ng/ml LPS for 4 days prior to determination of excess centrosome frequency. Results are shown in fold of increase, and each frequency was normalized to its respective control. Error bars, standard deviation from mean. Statistics: Two-tailed unpaired Student’s t-test (A), Χ^2^ test (B). *, p≤0.05; ns, not significant.

Chronic inflammation-associated signaling, which is activated by up-regulation of cytokines, is another characteristic of the tumor environment. IL-8 is a pro-inflammatory cytokine that regulates angiogenesis [21]. To determine if IL-8 promotes centrosome over-duplication in EC, we treated HUVEC with IL-8, which induced ERK phosphorylation in HMVEC (S3 Fig); however, these levels of IL-8 did not induce excess centrosomes (Fig 3A). To test more general effects of inflammation on centrosome over-duplication, HUVEC were treated with lipopolysaccharide (LPS), a potent pro-inflammatory agent that promotes secretion of a wide range of inflammatory mediators [22]. Consistent with the results of IL-8 treatment, LPS treatment did not induce significant increases in excess centrosomes in HUVEC (Fig 3B). These results indicate that IL-8 and LPS do not induce centrosome over-duplication in EC, suggesting that inflammatory mediators are not causative agents in generating excess centrosomes in EC.

### Hypoxia induces excess centrosomes in EC

**Fig 4:**
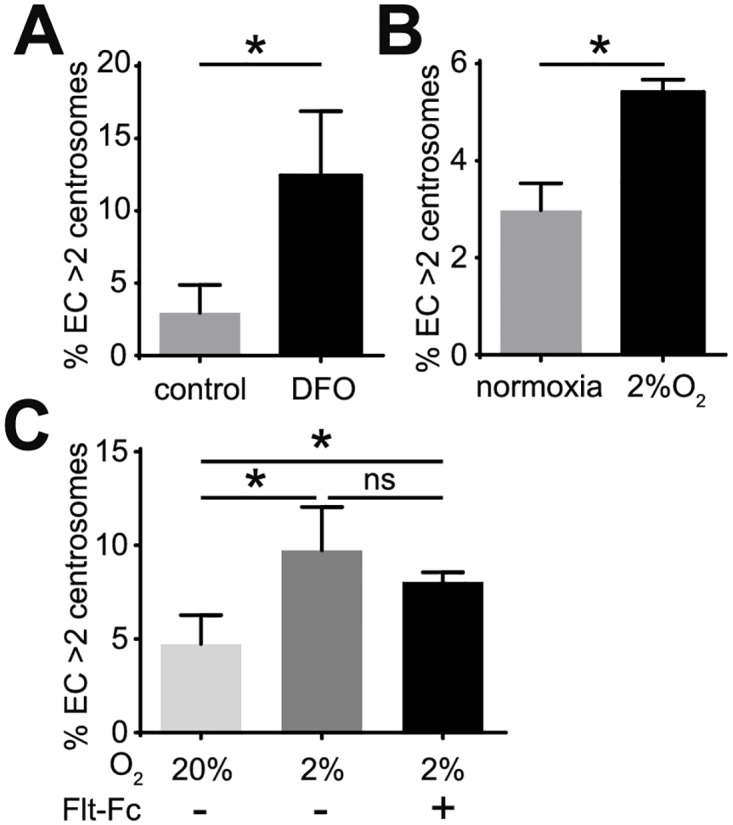
Hypoxia induces excess centrosomes in EC independent of cell-autonomous VEGF-A signaling. (A) Frequency of excess centrosomes in HUVEC after treatment with 100 μM hypoxic-mimetic agent desferrioxamine (DFO) for 4 days. (B) Frequency of excess centrosomes in HUVEC after 4 days of incubation in 2% oxygen. (C) Frequency of excess centrosomes in HUVEC after incubation in 20% or 2% oxygen for 4 days and indicated treatments. Error bars, standard deviation from mean. Statistics: two-tailed unpaired Student’s t-test. *, p≤0.05; ns, not significant.

In addition to the complex milieu of cytokines and growth factors, tumor environments are often hypoxic. To determine whether hypoxia induces excess centrosomes in EC, HUVEC were first treated with the oxygen chelating agent desferrioxamine (DFO), which mimics hypoxia in inducing HIF1α accumulation [23]. Treatment with DFO resulted in a 4-fold increase in the frequency of excess centrosomes compared to controls (Fig 4A). To further test our hypothesis, HUVEC were cultured in a 2–3% oxygen environment (hypoxia) for 4 days, then fixed and stained to assess the frequency of centrosome over-duplication. Hypoxic incubation led to translocation of HIF1α from the cytoplasm to the nuclear compartment of EC (S4A–S4C Fig), and also induced accumulation of HIF1α (S4D Fig), indicating the activation of hypoxia pathways. Incubation in 2% or 3% oxygen significantly promoted centrosome over-duplication compared to normoxic controls (Fig 4B, S4E Fig). These results indicate that a hypoxic environment is sufficient to induce excess centrosomes in EC.

Hypoxia up-regulates the production and release of pro-angiogenic cytokines such as VEGF-A in multiple tissues [15]. To determine whether hypoxia-induced centrosome over-duplication in EC requires VEGF-A signaling, HUVEC were incubated in hypoxic conditions with recombinant human soluble VEGF Receptor-1 (Flt-1)/Fc to block VEGF-A signaling. Flt-1/Fc treatment efficiently inhibited ERK phosphorylation induced by VEGF-A (S4F Fig), but was unable to rescue hypoxia-induced centrosome over-duplication (Fig 4C). This result suggests that hypoxia induces excess centrosomes in EC through VEGF-A-independent mechanisms.

### Inhibition of p53 signaling induces excess centrosomes in EC

**Fig 5:**
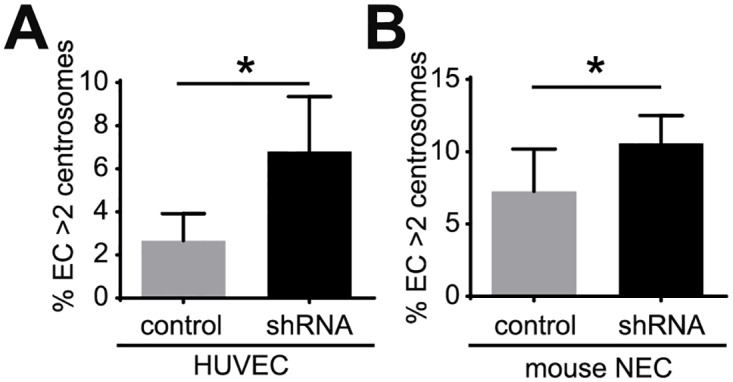
Down-regulation of p53 induces excess centrosomes in EC. (A) Frequency of excess centrosomes in HUVEC infected with human p53 shRNA. (B) Frequency of excess centrosomes in normal mouse endothelial cells (NEC) infected with mouse p53 shRNA. Error bars, standard deviation from mean. Statistics: two-tailed unpaired Student’s t-test. *, p≤0.05.

Loss or inactivation of p53 induces excess centrosomes in mouse embryonic fibroblasts [24]. Thus, we tested whether p53 attenuation leads to excess centrosomes in EC. A short-hairpin RNA (shRNA) was used to down-regulate p53 levels in HUVEC (S5A Fig), and HUVEC infected with p53 shRNA had an approximately 3-fold increase in the percentage of excess centrosomes (Fig 5A). Previous studies demonstrated that mouse tumor stromal cells, including mouse tumor EC, have an attenuated p53 response [25]. Therefore we asked whether down-regulation of p53 induced excess centrosomes in mouse EC by infecting immortalized normal mouse EC (NEC) [6] with p53 shRNA. Down-regulation of mouse p53 also induced excess centrosomes in NEC (S5B Fig, Fig 5B). These results suggest that down-regulation of p53 contributes to centrosome over-duplication in tumor EC.

## Discussion

We previously showed that high levels of the pro-angiogenic growth factors VEGF-A and bFGF promote excess centrosomes in EC [11]. However, the frequency of EC centrosome over-duplication, even with a combination of both VEGF-A and bFGF, was much less than that seen in primary isolates of tumor-derived EC [6], suggesting that other aspects of the tumor environment contribute to pathological centrosome over-duplication. Here we provide evidence that excess centrosomes in EC occur downstream of numerous tumor-related inputs. We found that the BMP ligands BMP2, BMP6 and BMP7 significantly induced centrosome over-duplication, while inflammatory mediators were ineffective. Hypoxia, which is associated with most solid tumors, induced excess centrosomes in EC through VEGF-A-independent mechanisms. Besides environmental factors, cell-autonomous perturbation of p53 also promoted excess centrosomes in EC. These findings suggest that multiple inputs contribute to the high frequency of tumor vessel-derived EC with excess centrosomes.

Elevated levels of some BMP ligands, similar to high levels of VEGF and FGF ligands, induce excess centrosomes in EC. Interestingly, VEGF and FGF signaling are mediated by VEGF receptor 2 and FGF receptor, respectively, which belong to the tyrosine kinase receptor family [26], whereas BMP signals through serine/threonine kinase receptors [27], suggesting that diverse signaling inputs promote centrosome over-duplication in EC. Our results also show ligand and cell type specificity of BMP in inducing excess centrosomes: BMP2, BMP6 and BMP7, but not BMP4, significantly induced excess centrosomes in HUVEC, whereas BMP2 and BMP6 did not significantly affect centrosome numbers in several other human primary EC.

BMP ligands initiate signal transduction by binding a hetero-tetrameric receptor comprised of two dimers of type 1 and type 2 receptors [20]. Among a group with specificity for TGFβ and/or BMP signaling, BMPR1A, BMPR1B and BMPR2 are specific to BMP ligands [20]. Here we show that knockdown of BMPR1A, but not BMPR1B or BMPR2, inhibits BMP-induced SMAD1/5 phosphorylation and centrosome over-duplication. BMPR1A is critically involved in BMP signaling, and BMPR1A knockout mice are embryonically lethal with severe heart valve and EC defects [28–30]. However, BMPR1B knockout are viable [31]. In line with the *in vivo* data, previous *in vitro* data showed that BMPR1A siRNA, but not BMPR1B siRNA, abrogates SMAD1/5 phosphorylation in human microvascular endothelial cells [32]. These results are consistent with our findings. Interestingly, BMPR2 knockdown did not inhibit SMAD activation or block BMP ligand-induced centrosome over-duplication, indicating possible redundancy of type 2 receptors in EC. This is also consistent with a previous finding that ablation of BMPR2 in pulmonary artery smooth muscle cells allows signaling through ActR2A and does not abolish BMP signaling [33].

Another prominent feature of the tumor environment is a chronic inflammatory response, which is mediated by infiltration of immune system cells [34]. Tumor inflammation is similar to inflammation associated with normal physiological processes such as wound healing [34]. Our results suggest that inflammatory mediators do not induce centrosome over-duplication in EC. Thus, despite being a hallmark of the tumor environment, chronic inflammation is likely not an input for centrosome over-duplication in tumor EC. This finding also suggests that during physiological inflammation, EC do not develop excess centrosomes, therefore maintaining a relatively normal phenotype and function.

Hypoxia upregulates the expression and secretion of growth factors, such as VEGF-A, in the tumor environment [35]. Here we show that hypoxia induces excess centrosomes in EC. However, although hypoxia-induced signaling up-regulates VEGF-A, which promotes centrosome over-duplication [11], our data suggest that hypoxia-induced excess centrosomes in EC are independent of EC-derived VEGF-A. This indicates that, if tumor EC undergo centrosome over-duplication as a result of up-regulated VEGF-A signaling in the tumor environment, the source of the ligand is likely the tumor cells or other non-endothelial stromal cells.

In addition to changes in the tumor environment, tumor EC may also acquire cell-autonomous perturbations that promote centrosome over-duplication. Previous studies showed that tumor stromal cells, including tumor EC, have attenuated p53 activation in response to stress stimulation [25], and p53 abnormalities have been linked with centrosome over-duplication. For example, mouse embryonic fibroblasts isolated from p53 knock-out mice possess multiple copies of functional centrosomes [24]. Here we show that reduced p53 levels induced excess centrosomes in EC, suggesting that cell autonomous p53 changes contribute to centrosome over-duplication in tumor EC.

Although up to 30% of primary tumor EC have excess centrosomes [6], our results indicate that no single environmental factor or down-regulation of p53 alone achieves such a high percentage of excess centrosomes in EC [11]. It is possible that *in vivo*, several inducing factors combine to achieve the high percentage of excess centrosomes in tumor EC. In summary, we show that multiple environmental inputs and attenuated p53 contribute to centrosome over-duplication in EC. This work contributes to our understanding of both normal and tumor angiogenesis, and provides potential insights for anti-angiogenic therapy.

## Supporting Information

S1 Fig Effects of BMP ligands on human primary EC. (A) HUVEC labeled with centrin-GFP (green) and stained with γ-tubulin (red) and pericentrin (blue). Different numbers (n) of centrosomes shown. Nuclear position was determined via DIC and marked with yellow dashed circles. White dashed squares indicate centrosomes shown in higher magnification to the right. (B) Frequency of excess centrosomes in HUVEC after treatment with 200 ng/ml of BMP4 for 4 days. (C-E) Frequency of excess centrosomes in HUAEC (C), HBMEC(D), or HMVEC-L (E) after treatment with 200 ng/ml of BMP2 or BMP6 for 4 days. Error bars, standard deviation from mean. Statistics: two-tailed unpaired Student’s t-test. ns, not significant. Scale bars: 10 μm unless indicated otherwise.
(TIF)

S2 Fig Validation of BMP receptor siRNAs. (A-C) Relative mRNA levels of BMPR1A (A), BMPR1B (B), or BMPR2 (C) in HUVEC treated with indicated siRNAs. Cells were collected 48 hr after siRNA treatment. Error bars: standard deviations from mean. Statistics: two-tailed unpaired. *, p≤0.05; ***, p≤0.001.
(TIF)

S3 Fig Elevated IL-8 activates ERK phosphorylation. HMVEC were treated with 200 ng/ml IL-8 or VEGF-A for indicated times, collected, and analyzed for phosphorylated ERK (pERK) and total ERK.
(TIF)

S4 Fig Hypoxia activates HIF1α and Flt-Fc blocks VEGF-A signaling. (A) HUVEC were treated with/without 100 μM CoCl_2_ for 4 hr before fixation and incubated with/without HIF1α primary antibody. Only nuclear HIF1α is shown (see Methods for details of mask). (B) Fluorescence intensity of nuclear HIF1α in HUVEC treated as indicated. (C) HUVEC were MeOH fixed immediately (lower panel) or after 30-min recovery in normoxia (top panel) post-hypoxic incubation, then stained for HIF1α (red) and DRAQ7 (DNA, green). (D) Western blot for HIF1α in HUVEC incubated in normoxia or 2% oxygen. (E) Frequency of excess centrosomes in HUVEC after incubation in 3% O_2_ for 4 days. (F) HUVEC were treated with VEGF-A (200 ng/ml) or VEGF-A plus Flt-Fc (1 ug/ml) for 20 min. Cell lysates were collected and blotted for phosphorylated ERK (pERK) and total ERK. Error bars, standard deviation from mean. Statistics: two-tailed unpaired Student’s t-test. *, p≤0.05; ***, p≤0.001. Scale bars: 20 μm.
(TIF)

S5 Fig Validation of p53 shRNA. HUVEC (A) or mouse normal endothelial cells (NEC) (B) were infected with viruses expressing human p53 shRNA or mouse p53 shRNA, respectively. p53 levels were detected by western blot 4 days after viral infection.
(TIF)

S6 Fig Original western blot images. Original full blot images corresponding to results in Fig 2E (A), S3 Fig (B), S4D Fig (C), S4F Fig (D), S5A Fig (E) and S5B Fig (F). Cropped areas for figures are shown in red boxes. Size markers are labeled in red.
(TIF)
