# Supplementary figures and images for: Tumor-Derived Factors and Reduced p53 Promote Endothelial Cell Centrosome Over-Duplication
Source: PLoS One. 2016 Dec 15;11(12):e0168334. doi: 10.1371/journal.pone.0168334 (PMC5158050; doi:10.1371/journal.pone.0168334)

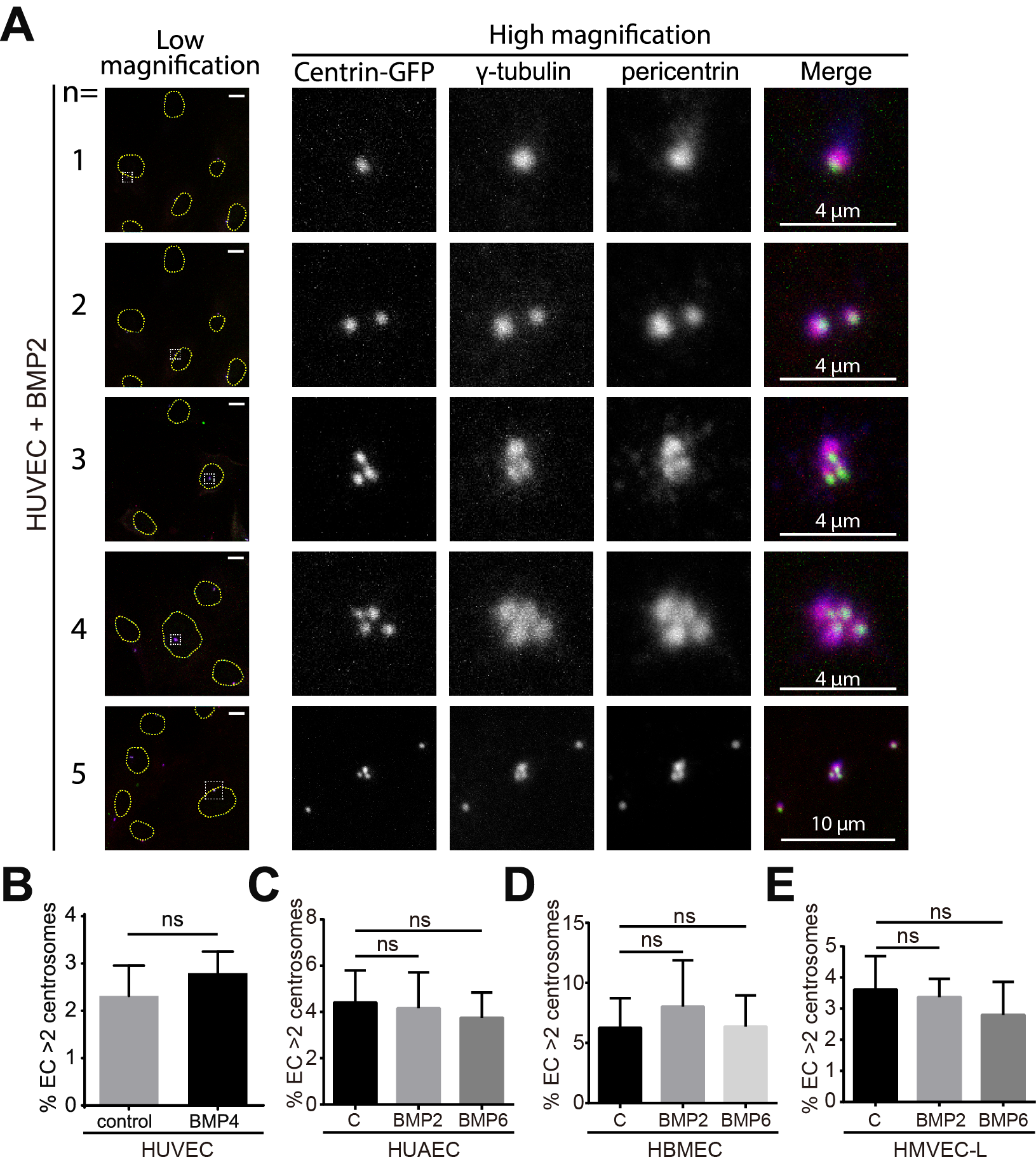

Supplement: S1 Fig — (A) HUVEC labeled with centrin-GFP (green) and stained with γ-tubulin (red) and pericentrin (blue). Different numbers (n) of centrosomes shown. Nuclear position was determined via DIC and marked with yellow dashed circles. White dashed squares indicate centrosomes shown in higher magnification to the right. (B) Frequency of excess centrosomes in HUVEC after treatment with 200 ng/ml of BMP4 for 4 days. (C-E) Frequency of excess centrosomes in HUAEC (C), HBMEC(D), or HMVEC-L (E) after treatment with 200 ng/ml of BMP2 or BMP6 for 4 days. Error bars, standard deviation from mean. Statistics: two-tailed unpaired Student’s t-test. ns, not significant. Scale bars: 10 μm unless indicated otherwise. (TIF) [file pone.0168334.s001.tif]

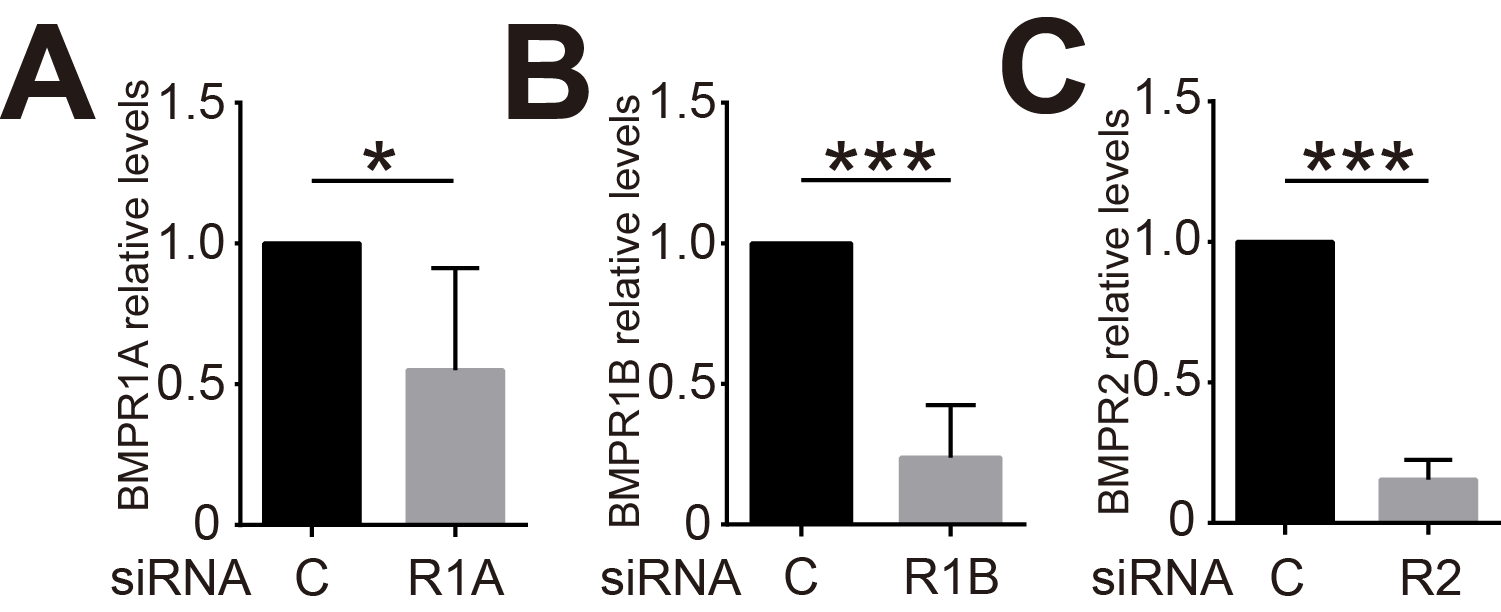

Supplement: S2 Fig — (A-C) Relative mRNA levels of BMPR1A (A), BMPR1B (B), or BMPR2 (C) in HUVEC treated with indicated siRNAs. Cells were collected 48 hr after siRNA treatment. Error bars: standard deviations from mean. Statistics: two-tailed unpaired. *, p≤0.05; ***, p≤0.001. (TIF) [file pone.0168334.s002.tif]

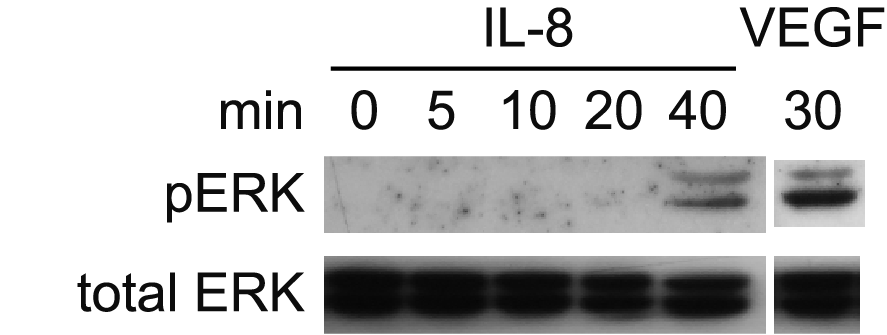

Supplement: S3 Fig — HMVEC were treated with 200 ng/ml IL-8 or VEGF-A for indicated times, collected, and analyzed for phosphorylated ERK (pERK) and total ERK. (TIF) [file pone.0168334.s003.tif]

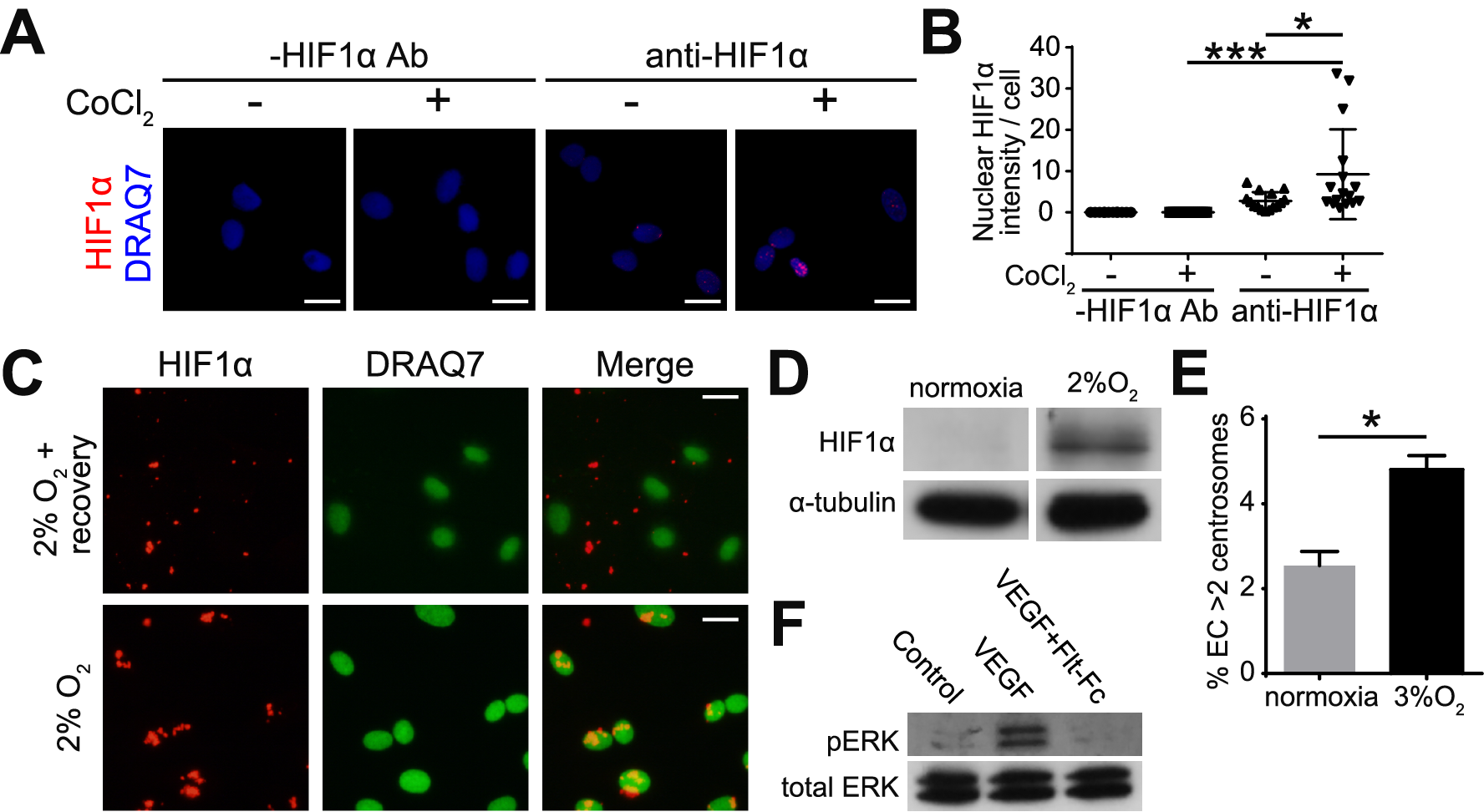

Supplement: S4 Fig — (A) HUVEC were treated with/without 100 μM CoCl2 for 4 hr before fixation and incubated with/without HIF1α primary antibody. Only nuclear HIF1α is shown (see Methods for details of mask). (B) Fluorescence intensity of nuclear HIF1α in HUVEC treated as indicated. (C) HUVEC were MeOH fixed immediately (lower panel) or after 30-min recovery in normoxia (top panel) post-hypoxic incubation, then stained for HIF1α (red) and DRAQ7 (DNA, green). (D) Western blot for HIF1α in HUVEC incubated in normoxia or 2% oxygen. (E) Frequency of excess centrosomes in HUVEC after incubation in 3% O2 for 4 days. (F) HUVEC were treated with VEGF-A (200 ng/ml) or VEGF-A plus Flt-Fc (1 ug/ml) for 20 min. Cell lysates were collected and blotted for phosphorylated ERK (pERK) and total ERK. Error bars, standard deviation from mean. Statistics: two-tailed unpaired Student’s t-test. *, p≤0.05; ***, p≤0.001. Scale bars: 20 μm. (TIF) [file pone.0168334.s004.tif]

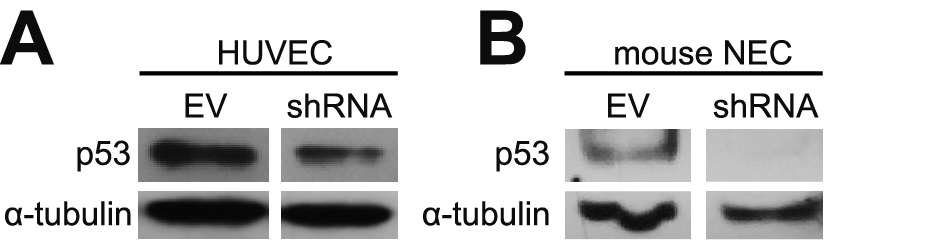

Supplement: S5 Fig — HUVEC (A) or mouse normal endothelial cells (NEC) (B) were infected with viruses expressing human p53 shRNA or mouse p53 shRNA, respectively. p53 levels were detected by western blot 4 days after viral infection. (TIF) [file pone.0168334.s005.tif]

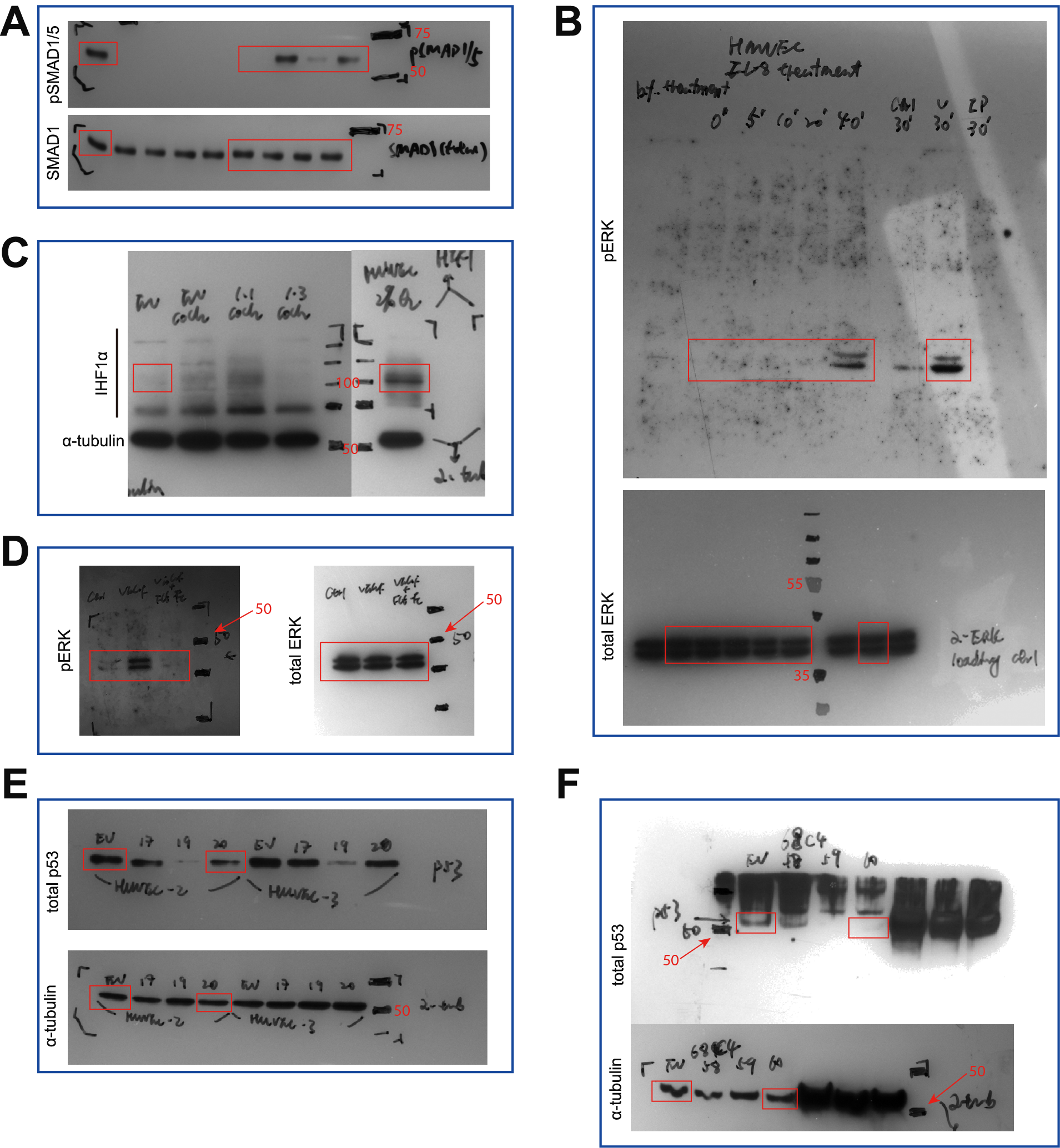

Supplement: S6 Fig — Original full blot images corresponding to results in Fig 2E (A), S3 Fig (B), S4D Fig (C), S4F Fig (D), S5A Fig (E) and S5B Fig (F). Cropped areas for figures are shown in red boxes. Size markers are labeled in red. (TIF) [file pone.0168334.s006.tif]
